# A plea for the development of an universally accepted modular tooth wear evaluation system

**DOI:** 10.1186/s12903-016-0309-6

**Published:** 2016-11-03

**Authors:** P. Wetselaar, A. Faris, F. Lobbezoo

**Affiliations:** Department of Oral Kinesiology, Academic Centre for Dentistry Amsterdam (ACTA), University of Amsterdam and VU University Amsterdam, MOVE Research Institute Amsterdam, Gustav Mahlerlaan 3004, 1081 LA Amsterdam, The Netherlands

**Keywords:** Diagnosis, Evaluation system, Index, Modular evaluation system, Tooth wear

## Abstract

**Background:**

Tooth wear is considered an increasing oral health problem. Due to its multifactorial nature, recognizing and diagnosing of tooth wear is difficult but nevertheless important. Over the years, a wide variety of evaluation systems has been developed, yet none of them is universally accepted. This has implications for both research and clinical practice.

**Discussion:**

This paper describes an in-depth analysis of four commonly used tooth wear evaluation systems, namely, the Eccles index, the Tooth Wear Index, the Lussi index, and the Basic Erosive Wear Examination. Comparing those systems revealed that despite several similarities, they differ considerably from each other. Notably, all four systems have their specific advantages and disadvantages. However, neither one of them meets all necessary characteristics of a hypothetical, broadly applicable tooth wear evaluation system. In fact, it is not realistic that a single system qualifies for all purposes (for example, diagnosing or monitoring individual patients, performing epidemiological studies, etc.).

**Summary:**

As a potentially feasible solution for this issue, the development of an evaluation system is recommended that consists of multiple, coherent modules, which cover different purposes.

## Background

Tooth wear is considered an increasingly prevalent oral health problem, which will mainly manifest in the future. It can be divided in the subtypes “mechanical wear” (attrition and abrasion), and “chemical wear” (erosion, or erosive tooth wear) [1]. Since nowadays most people grow old with their own dentition, tooth wear occurs more frequently [1]. Because tooth wear is always a multifactorial process, its diagnosis can be difficult [2]. Within dentistry, several widely accepted diagnostic tools for various pathologies are used (e.g., Decayed Missing Filled Teeth (DMFT) for caries and the periodontal screening index [3] for periodontal diseases). So far, however, no universally accepted diagnostic tool is available for the evaluation of tooth wear [4]. Most likely, this is due to the fact that a broadly applicable tooth wear evaluation system has not been developed yet. Ideally, such system should have the following characteristics: 1. being able to assess all subtypes of tooth wear, viz., mechanical wear and chemical wear; 2. being useful for research purposes (e.g., epidemiological and etiological assessments) as well as for clinical applications (e.g., screening, diagnosing, and monitoring); It is very important to have an evaluation system for tooth wear, that can be used for epidemiological purposes, because in that way, health care providers can estimate the need for dental care regarding tooth wear. Regarding individuals, since tooth wear is an irreversible process, it is important to screen for tooth wear on a regular basis, in order to prevent wear as much as possible and by doing this, to try to preserve dentitions as long as possible in an ageing population. 3. having well-defined, reproducible scoring criteria; and 4. being easy to use. It was this study’s first aim to perform an in-depth analysis of the characteristics of four commonly used tooth wear evaluation systems and to determine if, and if so, to what extent these systems show any one or more of the above-described characteristics of a hypothetical, broadly applicable evaluation system. The following systems will be analyzed: the Eccles index [5], the Tooth Wear Index, TWI [6], the Lussi index [7], and the Basic Erosive Wear Examination, BEWE [8]. The outcome of the analyses of the four systems will provide input for the second aim of this study, namely to formulate recommendations for the development of a broadly applicable tooth wear evaluation system for use in adults. By using the Eccles index, the Lussi index and the BEWE, chemical tooth wear is overestimated, because the two subtypes of tooth wear (chemical wear and mechanical wear) are summated.

### Selection of the most widely quoted tooth wear evaluation systems

The selection of the first three evaluation systems (i.e., the Eccles index, the TWI, and the Lussi index) was based on the frequency of them being quoted in the literature of the past four decades. This was based on several PubMed searches, the last one being performed on the 12th of September 2016. The used query was: “(tooth wear OR tooth surface loss OR attritive tooth wear OR abrasive tooth wear OR erosive tooth wear OR tooth attrition OR teeth attrition OR dental attrition OR tooth erosion OR teeth erosion OR dental erosion OR tooth abrasion OR teeth abrasion OR dental abrasion) AND (index OR indices OR scale OR scales OR measurement OR measurements OR grade OR grades OR grading system OR grading systems OR recording system OR recording systems OR evaluation system OR evaluation systems)”. The PubMed search yielded 114 different grading systems for the quantification of tooth wear, including adaptations of already existing systems. It was revealed that in research, the TWI is the most frequently used system, followed by the BEWE and the Lussi index respectively. Of importance for our selection was that these three systems were also used by other authors than those who developed the systems, while for the majority of the system, the use was restricted to the developers. Additionally, Margaritis and Nunn [4] described in their article in Table 3 their PubMed search of the most recently cited indices used in the assessment of erosive wear (years 2000–2013). They found a frequency of use of 13 times for the Lussi index, 11 times for the TWI, 6 times for the BEWE, and 5 times for the Eccles index respectively, when the target group was adults. No other indices with adults as the target group were included in their table. Hence, this is in line with our findings. The Eccles index is considered as one of the basic evaluation systems from which many others originate [9]. The TWI and the Lussi index are the two most commonly used tooth wear evaluation systems, and many other systems are developed based on them. The fourth system that was included, the BEWE, was recently introduced by a group of experts and is since then quoted frequently as well. Although the BEWE is a relatively new index and population-based studies are scarce, the amount of studies is increasing with recent reports from around the globe. The characteristics of these four evaluation systems are described below.

#### Eccles index

The Eccles index [5] consists of three classes, viz., I, II, and III (Table 1). Classes I and II apply to all surfaces, while Class III differentiates between various surfaces, namely facial surfaces as IIIa, lingual and palatal surfaces as IIIb, incisal and occlusal surfaces as IIIc, and severe multi-surface involvement as IIId.

**Table 1:** Eccles index [5]

Class	Description
I	Superficial lesions involving enamel only
II	Localized lesions involving dentin for less than 1/3 of the surface
III	Generalized lesions involving dentin for more than 1/3 of the surface
IIIa	Facial
IIIb	Lingual and palatal
IIIc	Incisal and occlusal
IIId	Severe multisurface involvement

#### Tooth Wear Index (TWI)

The Tooth Wear Index (TWI) [6] consists of a five-point ordinal scale. The descriptions of the various grades are shown in Table 2. The grading is identical for all teeth, but different criteria are present for the various surfaces, viz., for the buccal/lingual/occlusal, incisal, and cervical surfaces.

**Table 2:** Tooth Wear Index (TWI) [6]

Score	Description		
	Buccal/Lingual/Occlusal	Incisal	Cervical
0	No loss of enamel surface characteristics	No loss of enamel surface characteristics	No change in contour
1	Loss of enamel surface characteristics	Loss of enamel surface characteristics	Minimal loss of contour
2	Loss of enamel exposing dentine for less than 1/3 of the surface	Loss of enamel just exposing dentine	Defect <1 mm deep
3	Loss of enamel exposing dentine for more than 1/3 of the surface	Loss of enamel and substantial loss of dentine, but not exposing pulp secondary dentine	Defect 1–2 mm deep
4	Complete loss of enamel, or pulp exposure, or exposure of secondary dentine	Pulp exposure or exposure of secondary dentine	Defect >2 mm deep, or pulp exposure or exposure of secondary dentine

#### Lussi index

The Lussi index [7] consists of two ordinal scales for all teeth, viz., a four-point ordinal scale for the facial surfaces and a three-point ordinal scale for the occlusal/lingual surfaces. The descriptions of both scales are shown in Table 3.

**Table 3:** Lussi index [7]

Surface	Score	Description
Facial	0	No erosion. Surface with a smooth, silky-glazed appearance and absence of developmental ridges possible.
	1	Loss of surface enamel. Intact enamel found cervical to the erosion and concavity on enamel whose breadth clearly exceeds its depth, thus distinguishing them from tooth abrasion. Undulating borders of the lesions are possible. Dentin is *not* involved.
	2	Involvement of dentin for less than one half of the attacked area of the tooth surface.
	3	Involvement of the dentin for more than one half of the attacked area of the tooth surface.
Occlusal/lingual	0	No erosion. Surface with a smooth, silky-glazed appearance and absence of developmental ridges possible.
	1	Slight erosion. Rounded cusps, edges of restorations rising above the level of adjacent tooth surface, grooves on occlusal aspects. Loss of surface enamel. Dentin is not involved.
	2	Severe erosion, more pronounced signs than in grade 1. Dentin is involved.

#### Basic Erosive Wear Examination (BEWE)

The Basic Erosive Wear Examination (BEWE) [8] consists of a single four-point ordinal scale; the descriptions of the various grades are shown in Table 4.

**Table 4:** Basic Erosive Wear Examination (BEWE) [8]

Score	Description
0	No erosive tooth wear (no surface loss)
1	Initial loss of enamel surface texture
2^a^	Distinct defect, hard tissue loss (dentine) <50 % of the surface area
3^a^	Hard tissue loss >50 % of the surface area

^a^in scores 2 and 3, dentine often is involved

### Characteristics of a hypothetical, broadly applicable tooth wear evaluation system

A hypothetical, broadly applicable tooth wear evaluation system should have the following characteristics:

*Tooth wear subtypes:* being capable to evaluate both mechanical wear (attrition and abrasion) and chemical wear (erosion), and not only one subtype, since the subtypes rarely act alone.

*Assessment mode:* being applicable to all assessment modes, like chair side, dental casts, photographs, and/or scans, and not only for one or a few of them. This gives the clinician the possibility to choose the most appropriate assessment mode under the given clinical conditions.

*Purpose:* being capable of screening (both in the clinic and/or in epidemiological studies), diagnosing, and monitoring, and not for only one or two of these purposes.

*Clusters of teeth:* being suitable for both a partial assessment and a full assessment of the dentition. Examples of a partial assessment are grading only some key elements and grading of teeth per sextant. Also here, researchers and clinicians have the possibility to choose the most appropriate assessment mode.

*Surfaces:* being suitable for all possible (combinations of) surfaces, viz., occlusal, incisal, lingual/palatal/oral, buccal/labial/facial, cervical, occlusal/incisal, and/or non-occlusal/non-incisal surfaces. It should be noted that tooth wear rarely occurs on only one surface.

*Determining:* providing a good insight in the amount of tooth wear. To that end, the assessed grades should be noted separately. When the grades are added together, the severity can be camouflaged. Therefore, separate scores are to be preferred.

*Type of scale:* making use of clear scores, and not of descriptions that are open for multiple interpretations. Therefore, an ordinal scale is to be preferred over a nominal one.

*Direction of assessment:* using a combination of both surface area criteria (horizontal) and depth criteria (vertical, loss of clinical crown height), since early detection of tooth surface loss can better be determined with the former mentioned, while stages of more advanced tooth wear can better be determined with the latter mentioned.

*Amount of subscales/descriptions:* using as little subscales as possible, in order to make the use as straightforward as possible, and by this, avoiding the easy introduction of mistakes.

### Analysis of the four commonly used tooth wear evaluation systems

The characteristics of the four evaluation systems are described below and summarized in Table 5. In this paragraph, the descriptions are literal according to the mentioned authors.

**Table 5:** Characteristics of the four selected tooth wear evaluation systems (viz., Eccles index, Tooth Wear Index [TWI], Lussi index, and Basic Erosive Wear Examination [BEWE]), and of the hypothetical, broadly applicable, tooth wear evaluation system (h-TWES)

Characteristics	Evaluation systems				
	Eccles	TWI	Lussi	BEWE	h-TWES
Tooth wear subtypes					
tooth wear		x			x
mechanical					x
chemical	x		x	x	x
Possible overestimating chemical wear	x		x	x	
Assessment mode					
chair-side	x	x	x	x	x
dental casts		x		x	x
photographs	x	x		x	x
scans					x
Purpose					
screening				x	x
diagnosing	x	x	x	x	x
monitoring				x	x
Cluster of teeth					
partial assessment				x	x
full assessment	x	x	x		x
Surfaces					
occlusal	x	x	x	x	x
incisal	x	x	x	x	x
palatal/lingual/oral	x	x	x	x	x
buccal/labial/facial	x	x	x	x	x
cervical		x			
Determining					
cumulative				x	
separate	x	x	x		x
Type of scale					
ordinal	x	x	x	x	x
nominal					
Direction of assessment					
surface area	x	x	x	x	x
depth	x	x	x	x	x
Amount of Subscales and/or descriptions					
1				x	x
2			x		x
3		x			
4	x				

(x = meets the required characteristics)

#### Tooth wear subtypes

As indicated by the authors themselves, the Eccles index, the Lussi index, and the BEWE focus only on chemical wear (erosion) [5, 7, 8]. The TWI is the only index of the four selected evaluation systems that is applicable to all subtypes of tooth wear [6].

#### Assessment mode

All four evaluation systems (Eccles index, TWI, Lussi index, and BEWE) are suitable for chair side use [5–8]. For the use on dental casts, only the TWI and the BEWE are suitable [6, 8]. For the use on photographs, only the Lussi index is *not* suitable [7]. For none of the four evaluation systems, it is indicated whether they are suitable for the use with scans, but it must be noted that all systems were introduced before scans were broadly introduced.

#### Purpose

The BEWE is suitable for screening and monitoring [8]. For screening, a quick but not detailed overview of the dentition is needed. The Eccles index, the TWI, and the Lussi index are only suitable for diagnosing [5–7]. All teeth and surfaces are examined and recorded. This corresponds with the purpose of diagnosing, because this requires a detailed overview of the entire dentition.

#### Clusters of teeth

The Eccles index, the TWI, and the Lussi index examine and record all teeth [5–7]. The BEWE is the only index of the four selected evaluation systems that uses a partial assessment. The BEWE examines all teeth, but only the most severely affected tooth in a sextant is recorded [8].

#### Surfaces

The Eccles index grades the occlusal/incisal, facial, and lingual/palatal surfaces [5] (Table 1); the TWI the buccal/lingual/occlusal, incisal, and separately the cervical surfaces [6] (Table 2); the Lussi index the facial and occlusal/lingual surfaces [7] (Table 3); and the BEWE the buccal/facial, occlusal, and lingual/palatal surfaces [8] (Table 4).

#### Determining

The Eccles index [5], the TWI [6], and the Lussi index [7] determine the scores separately. Only the BEWE uses a cumulative score, whereby the six separate scores of each sextant are added to one cumulative score [8].

#### Type of scale

All four evaluation systems have an ordinal scale [5–8].

#### Direction of assessment

All four evaluation systems are based on a combination of grading the amount of the surface area that is involved and the depth of the tooth surface loss [5–8].

#### Amount of subscales/descriptions

The Eccles index can be considered as having four separate 3-point ordinal scales, namely for the facial surfaces (a), for the lingual and palatal surfaces (b), for the incisal and occlusal surfaces (c), and when multiple surfaces are involved at the same time (d). For each ordinal scale, the descriptions are identical, while the surfaces differ [5] (Table 1). The TWI has three separate 5-point ordinal scales, namely for the buccal/lingual/occlusal surfaces, for the incisal surfaces, and for the cervical surfaces. For each ordinal scale, the descriptions differ [6] (Table 2). The Lussi index consists of a 4-point ordinal scale for the facial surfaces and of a 3-point ordinal sale for the occlusal/lingual surfaces. The description of grade 0 is identical for both scales, while for the other grades (1, 2, and 3) the descriptions differ [7] (Table 3). The BEWE has only one 4-point ordinal scale, the descriptions of which are used for all surfaces [8] (Table 4).

## Discussion

The analyzed four evaluation systems (viz., Eccles index, TWI, Lussi index, BEWE) have several similarities, but on the other hand, they differ considerably and do not complement each other (Table 5). None of the four systems shows all characteristics of a hypothetical, broadly applicable tooth wear evaluation system (Table 5). Below, those characteristics will be discussed.

### Tooth wear subtypes

It is often stated that the subtypes of tooth wear hardly exist separately [2, 10]. Also the developers of the four selected tooth wear evaluation systems mentioned that in their articles. For example, Eccles [5] stated:” *Loss of tooth substance as a result of erosion is frequently made worse by abrasion, so that it may be impossible to make a clear distinction between the two conditions” and “Attrition, wear due to opposing teeth, will also exacerbate the effects of erosion.”* [7] stated for other than facial surfaces: “*Erosion, attrition and abrasion are difficult to distinguish in their initial stages,”* and *“When erosion is present, abrasion and attrition can cause it to increase.”.* While the BEWE was originally developed for the assessment of erosion [8], the system was later described as “a proposed system for screening tooth wear” [11]. Smith and Knight [6] designed a tool for diagnosing all three subforms of tooth wear and their combinations. They stated: *“Together with the fact that there is often a combined cause, the simple term tooth wear is proposed to embrace all three conditions plus their combination.*” [6]. Since it can be concluded that the different subtypes of tooth wear are difficult to differentiate, it can be stated that the Eccles index, the Lussi index, and the BEWE do measure different types of tooth wear, and not only one of the subtypes.

### Assessment mode

All four evaluation systems were designed to assess tooth wear chairside. For three of them, the authors stated that the use of photographs was possible as well [5, 6, 8], while for two of them the authors stated that the use of dental casts was a third option [6, 8]. Lussi and coauthors only mention the chairside use [7]. There is evidence that several evaluation systems can be used both on dental casts and on photographs [12] as well as both clinical and on casts [13]. Although this evidence concerns other evaluation systems than the four described in this article, namely the Visual Erosion Dental Examination (VEDE) [14] and an occlusal and non-occlusal Tooth Wear Grading System [13], it is an interesting finding. In the future, when dentals casts are possibly replaced by intraoral scans, it must be tested if the use of scans is as accurate as assessments performed chairside, on casts, and on photographs.

### Purpose

All four evaluation systems were designed, according to the authors, to diagnose (erosive) tooth wear. It goes without saying that only grading (quantifying) the amount of tooth surface loss is nowhere near a proper diagnosis [2]. Also qualification, recognizing the clinical signs of tooth wear [15], a proper oral history [2], and perhaps saliva tests are required [10]. Nevertheless, quantification is necessary, and for an individual patient, a thorough assessment of the present amount of tooth wear must be performed. The four evaluation systems all seem to be appropriate for this purpose. For screening (of individual patients and in epidemiological studies), a simple and short assessment is preferred. For this purpose, a partial assessment is appropriate. The BEWE uses a partial assessment by only writing down the surfaces/teeth with the highest amount of tooth surface loss per sextant [8]. For a more detailed assessment per individual, the BEWE grading could be used to assess all teeth and surfaces. The other way around is perhaps also possible, namely using the Eccles index, the TWI, or the Lussi index to assess the amount of tooth surface loss of all elements and surfaces (as aimed by the developers), but then only writing down the worst affected surfaces/teeth per sextant. It should be tested if the evaluation systems can be used in this manner. Concerning monitoring, it first needs to be clarified what is meant by this term. The authors of the BEWE consider monitoring a management strategy, that follows the effects the preventive measurements after counseling, and a guidance towards other treatment modalities (e.g., restorative treatment) [8]. In general, monitoring is considered a technique to measure progression. The authors of the BEWE mention that their system is not suitable for monitoring, because the distinction between the various levels is too crude [8]. The authors of the other three indices do not mention monitoring in their respective articles [5–7]. It is obvious that these evaluation systems fail twofold in this respect: I. the distinction between the levels is too crude, and II. for more advanced stages of tooth surface loss, there are no grades described. Regarding the too crude distinctions (I.), adaptations for all the four evaluation systems could be the introduction of intermediate steps (for example separating a score 2 into sub-scores 2a, 2b, and 2c). Regarding the more advanced stages of tooth surface loss (II.), for example for the TWI, this was already mentioned and adapted by Donachie & Walls [16]. They extended the TWI with a score 5, so changing the original 5-point ordinal scale into a 6-point ordinal scale [17].

### Clusters of teeth

The Eccles index, the TWI and the Lussi index use full assessment, which means, all teeth and all surfaces are graded [5–7]. Only the BEWE uses a partial assessment, by which the authors mean that all teeth and surfaces are assessed, but only the surface/tooth with the highest grade per sextant is recorded [8]. In fact, this is a full assessment as well, but with partial recording. During a real partial assessment, only so called key elements are graded and noted. An example of this way of assessing is the simplified erosion partial recording system (SEPRS) by Hasselkvist and coauthors, using only four permanent surfaces [18]. For screening purposes (on a patient level and/or in epidemiological studies), a partial assessment or a full assessment with a partial recording could be sufficient. For diagnosing, a full assessment is a necessity, while for monitoring (i.e., measuring progression) a full assessment is required.

### Surfaces

All four evaluation systems grade all surfaces of the clinical crown [5–8]. Only the TWI also grades the cervical surfaces [6]. Although it is clear that the surface structure of the roots is different from that of crowns, the necessity to grade the cervical surfaces separately can be discussed. The other three evaluation systems grade the cervical areas as part of the non-occlusal/non-incisal surfaces (buccal/lingual/palatal/oral). Since for the cervical areas in the TWI an extra ordinal scale with different description is necessary, this can make the use of the TWI unnecessarily difficult.

### Determining

The Eccles index [5], the TWI [6], and the Lussi index [7], determine the scores separately. Only BEWE uses a cumulative score, whereby the six separate scores of the sextants are combined into a single cumulative score [8]. Although it is highly attractive to give a tooth wear patient only one score, this not realistic. When adding up the different scores and calculating a cumulative score, the risk is that higher grades of tooth wear in one of the sextants are masked by lower ones in the other sextants. For example, a patient with severe tooth wear on the palatal surfaces of the second sextant, perhaps needs restorative treatment. When the scores remain separate, the clinician is alert, but when the scores are combined, the clinician can overlook the necessity for restorative treatment.

### Type of scale

All four evaluation systems have an ordinal scale (Table 5). A nominal scale is only useful for determining which subtype of tooth wear is present, based on clinical signs [15].

### Direction of assessment

All four evaluation systems are based on a combination of grading the amount of the surface area (horizontal) that is involved and the depth of the tooth surface loss (vertical, loss of clinical crown height). For assessing the early stages of tooth wear, grading the surface area is necessary. When the tooth surface loss progresses, grading in depth is necessary.

### Amount of subscales/descriptions

For an easy-to-use evaluation system, with well-defined, reproducible scoring criteria, it is useful that the system only has one ordinal scale, like the BEWE [8] has. Nevertheless, the occlusal/incisal surfaces differ that much from the non-occlusal/non-incisal surfaces, that this is not possible when the index is used for monitoring or diagnosing. The most important difference between these two groups of surfaces is the loss of clinical crown height, which is the case on occlusal/incisal surfaces, while on non-occlusal/non-incisal surfaces the clinical crown length is not affected.

#### Recommendations for the development of a broadly applicable tooth wear evaluation system

After analyzing the four commonly used tooth wear evaluation systems in depth, comparing their characteristics towards an hypothetically broadly applicable tooth wear evaluation system, the following recommendations for the development of a broadly applicable tooth wear evaluation system are raised. It must be concluded that, taking into account all the different characteristics, only one single evaluation systems is not realistic. Therefore, the development of a modular evaluation system seems to be a workable solution. Below, a possible solution is proposed, based on the above described analyses of the four evaluation systems. In a modular evaluation system, the following modules should be included: 1. A screening module (both for epidemiological studies and for screening individual patients in the clinic) (Table 6); and 2. A module for diagnosing or monitoring individual patients, for which both occlusal/incisal and non-occlusal/non-incisal (finer grained) evaluation systems are needed (Table 7). Regarding the management of an individual patient, we think that during every recall appointment, assessment of tooth wear is a necessity. Because a lack of time can be a factor, the screening module is suitable. When the health care provider want to monitor the progress of the tooth wear, the finer grained assessment can be performed during the recall sessions. Also, when a treatment plan for an individual patient is needed, one can assess the tooth wear in detail. One must realize that by the proposed modular evaluation system only quantification is performed and no qualification is done. Concerning the screening module, the proposal is a 4-point ordinal scale as shown in Table 6. One can discuss about the cluster of teeth, as well as about the surfaces. One can assess only some key elements, per sextant, or all elements. One can assess only occlusal/incisal or also non-occlusal/non-incisal. Until agreement is reached, every individual researcher and/or dental clinician can make his/her own decision, based on the specific goal of the assessment. Concerning the diagnosing/monitoring module, the proposals are a 6-point ordinal scale for the occlusal/incisal surfaces, and a 5-point ordinal scale for the non-occlusal/non-incisal surfaces. These ordinal scales are based on the descriptions of the four analyzed tooth wear evaluation systems [5–8], and on the tooth wear evaluation systems as described by Lobbezoo and Naeije [19] and Wetselaar and coworkers [13]. The modular evaluation system consists of three different ordinal scales. The descriptions for these three scales are similar for scores 0 and 1. For score 2, a different description exists; in the screening module the distinction in clinical crown height is ≤1/2, while in the finer grained diagnosing/monitoring scale the cutoff point is ≤1/3. The same applies to score 3; in the screening module the distinction in clinical crown height is >1/2, while in the finer grained diagnosing/monitoring scale the cutoff point is >1/3 but <2/3. Score 4 is similar for the occlusal/incisal surfaces and the non-occlusal/non-incisal surfaces. Score 5 only exists for the occlusal/incisal finer grained module. By this, the amount of scales and descriptions are as limited as possible.

**Table 6:** Modular evaluation system, module for screening, both occlusal/incisal and non-occlusal/non-incisal [4-point ordinal scale]

Score	Description
0	No (visible) wear
1	Wear within the enamel
2	Wear with dentin exposure - horizontal less than 50 % of the area *or* - loss of clinical crown height ≤1/2
3	Wear with dentin exposure - horizontal more than 50 % of the area *or* - loss of clinical crown height >1/2

**Table 7:** Modular evaluation system, module for diagnosing/monitoring occlusal/incisal [6-point ordinal scale], and non-occlusal/non-incisal [5-point ordinal scale]

Score	Description -occlusal/incisal	Description -non-occlusal/non-incisal
0	No (visible) wear	No (visible) wear
1	Wear within the enamel	Wear within the enamel
2	Wear with dentin exposure -horizontal less than 50 % of the area *or* -vertical loss of clinical crown height ≤1/3	Wear with dentin exposure -less than 50 % of the area
3	Wear with dentin exposure -horizontal more than 50 % of the area *or* -vertical loss of clinical crown height >1/3 but ≤1/2	Wear with dentin exposure -more than 50 % of the area
4	Wear with dentin exposure -horizontal complete loss of enamel *or* -vertical loss of clinical crown height >1/2 but ≤2/3	Wear with dentin exposure -complete loss of enamel *or* -pulp exposure
5	Wear with dentin exposure -horizontal complete loss of enamel *or* -vertical loss of clinical crown height >2/3	

## Conclusion

Four of the most commonly used evaluation systems have been analyzed in this paper to formulate recommendations for the development of a universally and broadly applicable “ideal” tooth wear evaluation system. The analyses reveal that none of the systems is suitable as a universal evaluation system in its current form. In addition, the study reveals that it is not feasible that a single evaluation qualifies for all purposes as a universal evaluation system should do. As the purpose determines which cluster of teeth and which surfaces should be examined, and since each purpose dictates a different examination, it is not realistic to have a single multi-purpose universal evaluation system for clinical use.

A potentially feasible solution could be a modular evaluation system that consists of multiple modules. It is stated that the modular system must be able to grade all subtypes of tooth wear, be applicable for all mentioned assessment modes, all purposes, all surfaces, and all directions of assessment. The types of scales are ordinal, the scores remain separate. One can choose for partial or full assessment, and the amount of subscales or descriptions must be as limited as possible.

More research is required to explore the feasibility of such a modular evaluation system. The dental community must take its responsibility to reach an agreement upon this topic.

## Abbreviations

BEWE: Basic erosive wear examination
DMFT: Decayed missing filled teeth
SEPRS: Simplified erosion partial recording system
TWES: Tooth wear evaluation system
TWI: Tooth wear index
VEDE: Visual erosion dental examination
